# Study on Preparation and Performance of Foamed Lightweight Soil Grouting Material for Goaf Treatment

**DOI:** 10.3390/ma16124325

**Published:** 2023-06-12

**Authors:** Zhizhong Zhao, Jie Chen, Yangpeng Zhang, Tinghui Jiang, Wensheng Wang

**Affiliations:** 1 Guangxi Baining Expressway Co., Ltd., Nanning 533800, China; liuzyjlu2019@163.com; 2 Guangxi Key Lab of Road Structure and Materials, Nanning 530007, China; cj_engineering@163.com (J.C.); jth1949@outlook.com (T.J.); 3 Guangxi Hetian Expressway Co., Ltd., Nanning 530022, China; 4 Guangxi Transportation Science and Technology Group Co., Ltd., Nanning 530007, China; 5 School of Traffic and Transportation Engineering, Changsha University of Science and Technology, Changsha 410114, China; 6 College of Transportation, Jilin University, Changchun 130025, China

**Keywords:** goaf treatment, grouting material, foamed lightweight soil, water–solid ratio

## Abstract

The harm goafs and other underground cavities cause to roads, which could lead to secondary geological hazards, has attracted increased attention. This study focuses on developing and evaluating the effectiveness of foamed lightweight soil grouting material for goaf treatment. The study examines the foam stability of different foaming agent dilution ratios by analyzing foam density, foaming ratio, settlement distance, and bleeding volume. The results show that there is no significant variation in foam settlement distance for different dilution ratios, and the difference in foaming ratio does not exceed 0.4 times. However, the bleeding volume is positively correlated with the dilution ratio of the foaming agent. At a dilution ratio of 60×, the bleeding volume is about 1.5 times greater than that at 40×, which reduces foam stability. Furthermore, an appropriate amount of sodium dodecyl benzene sulfonate improves both the foaming ability of the foaming agent and the stability of the foam. Additionally, this study investigates how the water–solid ratio affects the basic physical properties, water absorption, and stability of foamed lightweight soil. Foamed lightweight soil with target volumetric weights of 6.0 kN/m^3^ and 7.0 kN/m^3^ meet the flow value requirement of 170~190 mm when the water–solid ratio ranges are set at 1:1.6~1:1.9 and 1:1.9~1:2.0, respectively. With an increasing proportion of solids in the water–solid ratio, the unconfined compressive strength initially increases and then decreases after 7 and 28 days, reaching its maximum value when the water–solid ratio is between 1:1.7 and 1:1.8. The values of unconfined compressive strength at 28 days are approximately 1.5–2 times higher than those at 7 days. When the water ratio is excessively high, the water absorption rate of foamed lightweight soil increases, resulting in the formation of connected pores inside the material. Therefore, the water–solid ratio should not be set at 1:1.6. During the dry–wet cycle test, the unconfined compressive strength of foamed lightweight soil decreases, but the rate of strength loss is relatively low. The prepared foamed lightweight soil meets the durability requirements during dry–wet cycles. The outcomes of this study may aid the development of enhanced approaches for goaf treatment using foamed lightweight soil grout material.

## 1. Introduction

The mining industry has been a crucial element of human civilization for millennia. Nevertheless, the issue of goaf treatment has become increasingly significant with the advancement of mining activities [1,2]. The goaf—the excavated space left behind after minerals are extracted—poses substantial risks to the environment, as well as to the safety of mining operations and transport, if not adequately treated [3,4,5]. Therefore, the development of an efficient goaf treatment method is imperative to secure the sustainable growth of the transportation infrastructure.

Researchers from both domestic and international institutions have taken an avid interest in studying the effects of underground cavities, such as goafs, on highways, due to their potential to cause significant damage [6,7]. With the swift growth of China’s expressway construction, certain highways have been affected to differing degrees by secondary geological hazards stemming from coal mining subsidence in recent years. As a result, some scholars have initiated investigations into goaf impacts. Cao et al. discussed research on detecting goafs in mines, emphasizing the significance of precision exploration techniques and the adoption of established technology, such as 3D seismic exploration and transient electromagnetic method, for identifying presumed mining areas [8]. Their study also introduces the usage of 3D laser scanning technology to visualize concealed mined-out areas and analyze their formation mechanisms. Zhang et al. [9] examined the stress on the center of a goaf roof in a gypsum mine affected by faults, concluding that the mining stope’s structural parameters are reasonable but faults can cause subsidence. To evaluate the stability of surface structures, accurate detection approaches for deserted goafs are indispensable. A combination of incremental, conventional, and seismic methodologies was employed to locate an abandoned goaf on China’s Mu Shi expressway [10]. Han et al. analyzed the excavation stability and reinforcement measures of a cutting slope with a collapsed goaf roadway and mining face, finding that the collapsed mining face was the main factor affecting stability and proposing specific slope ratios for excavation as well as reinforcing methods [3]. In general, the technology for building highways in goaf regions remains in its early stages of development, with no established systematic theories or technological frameworks in place. As a result, the stability and strengthening of goaf roadways are crucial areas of inquiry.

After coal seams are extracted, abandoned goaf areas are created that disrupt the original state of equilibrium in the rock formations and generate secondary stresses [11,12]. As soon as the stress accumulates and exceeds the critical threshold of the surrounding rock, the goaf will become distorted and collapse, resulting in disasters such as roof fractures and collapse of overlying strata. This poses a risk to lives and properties while simultaneously leading to wastage of land resources. Moreover, uneven settlements of embankments in road construction may cause the roadbed and pavement structures to crack. Currently, reinforcement is the most effective method for treating goaf areas [13,14]. Grouting reinforcement is a widely employed treatment measure for both domestic and international highway goaf zones [15,16,17]. It involves injecting a configured slurry material into the rock and soil of the goaf area using appropriate equipment. The slurry has fluidity when it is freshly prepared. Once it reaches voids, cracks, or pores in the rock and soil, it solidifies and hardens gradually into a robust consolidation body that adheres to the original rock and soil, forming a whole. This technique improves the bearing capacity, impermeability, and deformation control of the rock and soil. Wang et al. proposed a modified theoretical formula for seepage grouting in goaf foundations that takes into account the fracture distribution characteristics and superposition effect of porous grouting. The formula was validated through laboratory tests and has great engineering significance, as it aids in designing optimal spacing between grouting holes to reduce residual deformation and activation deformation in goaf foundations [18]. When selecting grouting materials for reinforcement projects, it is important to consider their impact on the reinforcement effect and cost [19]. Researchers from around the world have conducted extensive research on grouting materials, mainly focusing on cement–clay slurry, single-cement slurry, cement–fly ash slurry, cement–water glass slurry, and other materials [20]. Using conventional grouting materials to fill goaf areas makes it difficult for the slurry to quickly accumulate, results in significant loss of slurry, and leads to low stone formation rates, thereby increasing the cost of treatment. New materials and technologies, including recycled rubber crumbs [21,22] and steel fiber [23], have been developed to enhance the performances of concrete. Additionally, temperature and humidity greatly influence the performance of grouting materials, particularly their dry-wetting resistance, which is highly relevant in actual engineering [24]. Lightweight fillers, compared to traditional grouting materials, offer several benefits such as being lightweight, possessing high strength and good seismic performance, being environmentally friendly and conserving resources [25,26,27]. Foamed lightweight soil grouting material is widely used in the field of civil engineering due to its excellent performance, including its high strength, low thermal conductivity, and good workability [28,29,30]. In recent years, researchers have applied this material to the goaf treatment field, providing advantages over traditional methods such as backfilling with solid materials or filling with water. It can be easily injected into the goaf through pipelines, filling up the space effectively, and thereby greatly reducing the risk of surface subsidence caused by goaf collapse [31,32].

This study aims to investigate the development and effectiveness of a foamed lightweight soil grouting material for the treatment of goaf. The prepared material will be used for the goaf treatment in the Guangxi Province, China. The study involves preparing the foamed lightweight soil grouting material for goaf treatment and discussing its performance. Firstly, the foam stability of different foaming agent dilution ratios is analyzed by testing the density, foaming ratio, settlement distance, and bleeding volume. Furthermore, the influence of the water–solid ratio on the basic physical properties of the material is investigated. Additionally, the water absorption and stability of the material are studied.

## 2. Materials and Methods

### 2.1. Raw Materials

This study investigates the use of cement, foaming agents, and water as the primary raw materials for foamed lightweight soil grouting material in goaf treatment. Cement acts as the main cementitious material in the preparation process, providing strength to the foamed lightweight soil through hydration and hardening reactions. Due to the large number of air bubbles in the foamed lightweight soil, the resulting material has relatively lower strength compared to ordinary soil. To ensure that the strength of the material meets project requirements, higher-quality cement is typically required compared to ordinary soil. Ordinary Portland Cement with a grade of 42.5 is selected for this experimental study due to its high early strength, fast setting time, and high density, which make it an effective cementitious material. The basic performance of the cement used in this study is listed in Table 1; it meets the requirements of “Common Portland Cement” (GB 175-2007) specifications in China.

In the preparation of foamed lightweight soil grouting material, a foaming agent is a necessary raw material that significantly affects the resulting material’s various properties. For this study, a composite foaming agent obtained from Henan Huatai Cement Technology Co., Ltd (Zhengzhou, China) was selected, as it meets the requirements of Chinese standard “Technical Specification for Design and Construction of Cast-in situ Foamed Light-weight Soil Subgrade” (TJG F1001-2001). Section 3.1 provides details on the foaming agent’s specific performance indices, such as foam density, settlement distance, foaming ratio, and bleeding volume at various dilution ratios.

Water is also a significant constituent of foamed lightweight soil grouting material, with its quality and consumption directly impacting the resulting material’s properties. To avoid impacts on foam quality and material properties, blending water must be free from impurities such as oil or organic matter. Excessive impurities in blending water can negatively impact foam quality by reducing the effectiveness of foaming agents. Furthermore, harmful impurities in water can erode the cement paste when mixed, thereby influencing the properties of the resulting foamed lightweight soil grouting material. Additionally, water consumption affects the material’s physical and mechanical properties and its durability. Therefore, to guarantee the quality of foamed lightweight soil grouting material, strict control of water quality and consumption during the preparation process is necessary. Moreover, the maximum particle size of soil aggregate should not exceed 5 mm, in accordance with the Chinese standard “Technical Specification for Cast-in situ Foamed Lightweight Soil” (CECS 249: 2008).

### 2.2. Preparation Method of Foamed Lightweight Soil Grouting Material

In this study, the prefabricated foam mixing method is used to prepare the foamed lightweight soil grouting material. The detailed preparation process of foamed lightweight soil grouting material is described as follows:

1. Step 1: According to the mix proportion scheme, the corresponding cement and water are weighted and then added to the mixer for the first mixing;
2. Step 2: According to the corresponding dilution ratio, the water and foaming agent are weighted for dilution and added into the foaming machine. The foaming agent diluent is introduced into air under pressure, thus generating a foam group;
3. Step 3: A certain amount of foam is weighted using an electronic balance, which is poured into the mixer. Then, it is mixed with the cement paste for 2 min;
4. Step 4: After the mixture is stirred evenly, the physical performance tests such as flow value, wet weight, etc. are conducted. In addition, the prepared foamed lightweight soil mixture is placed in the mold for manual vibration and molding. After the mold is removed, it is cured to the specified curing age according to the standard method, and then the subsequent performance test is carried out.

**Figure 1:**
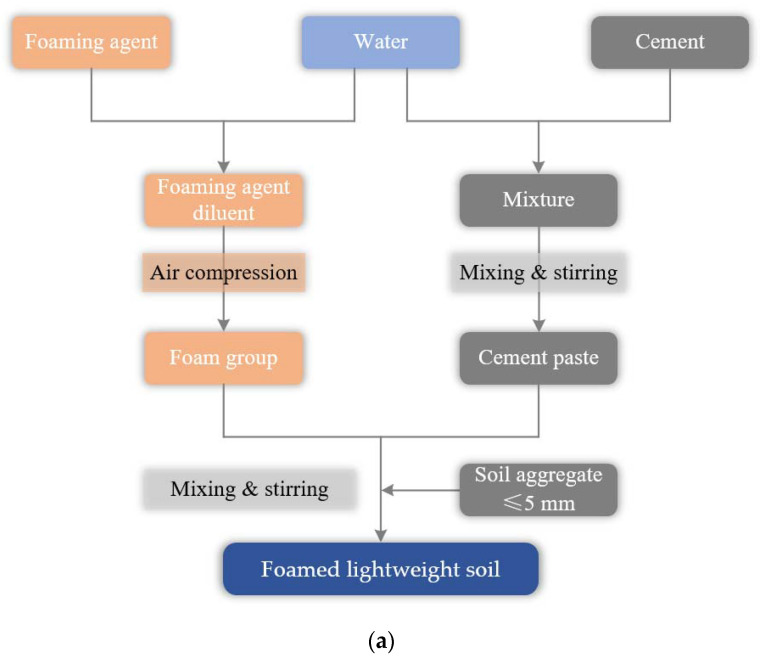

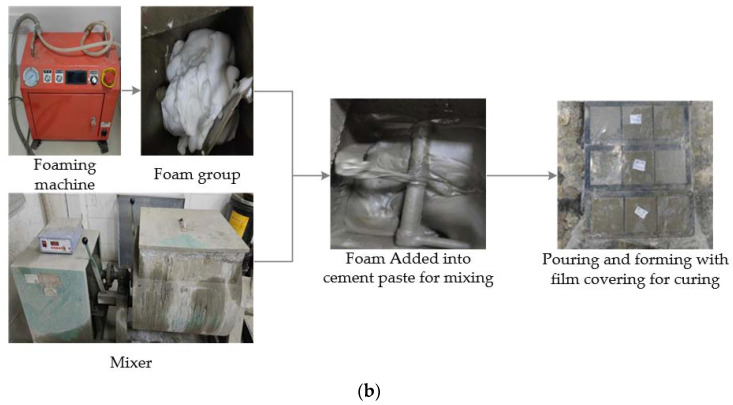
The preparation process of foamed lightweight soil grouting material: (**a**) Preparation process flow; (**b**) preparation process procedure.

The main instruments used to prepare the foamed lightweight soil grouting material are a foaming machine and a mixer. The preparation process flow and procedure of foamed lightweight soil grouting material are shown in Figure 1.

The foaming machine and mixer are the primary instruments used to prepare foamed lightweight soil grouting material. The process flow and procedure for this preparation are illustrated in Figure 1. In accordance with the Chinese standard “Specification for Design of Highway Subgrades” (JTG D30-2015), the minimum and maximum construction wet densities of foamed lightweight soil grouting materials should be greater than 500 kg/m^3^ and less than 1100 kg/m^3^, respectively. To assess the effects of the water–solid ratio on the resulting material, water–solid ratios were varied between 1:1.6~1:2.0 for target volumetric weights of 6.0 kN/m^3^ and 7.0 kN/m^3^. The specific mix proportion scheme is detailed in Table 2.

### 2.3. Experimental Methods

#### 2.3.1. Stability Test of Foam

Referring to the Chinese standard “Technical Specification for Foamed Concrete Application on Highway” (DB33/T 996-2015), the density and foaming ratio of foam could be tested. The test process was repeated three times for parallel experiments and the average value was taken. The foaming ratio (*M*) could be calculated as shown in Equation (1):

*M* = *V*/(*m*_2_ − *m*_1_)/*ρ*_0_, (1)

in which *ρ*_0_ is the density of the foam solution (taken as 1.0 g/cm^3^); *m*_1_ is the mass of the empty dry stainless steel measuring cup (g); *m*_2_ is the total mass of the stainless-steel measuring cup and foam (g); *V* is the volume of the stainless-steel measuring cup (cm^3^)

**Figure 2:**
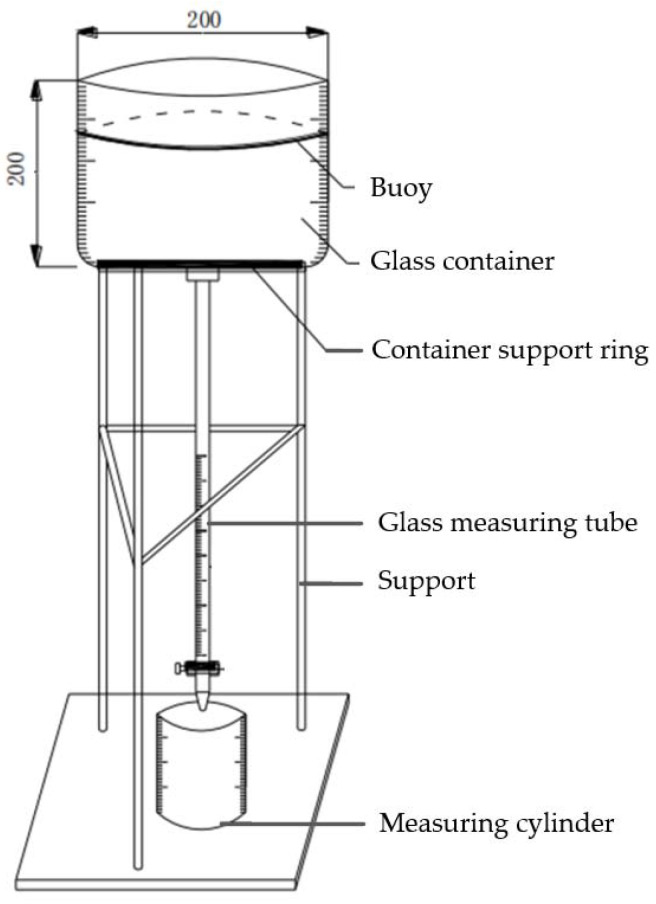
The testing device of settlement distance and bleeding volume of foam.

The effectiveness of foamed lightweight soil depends on the quality of the foaming agent; therefore, this study subjected the foaming agent to a series of performance tests and optimized the preparation parameters. Settlement distance pertains to the foam settling distance after standing still for one hour, while bleeding volume refers to the amount of water that leaks out due to foam rupture during the same period. Both measurements were taken for 1 L of foam. Similarly, in accordance with the Chinese standard “Technical Specification for Foamed Concrete Application on Highway” (DB33/T 996-2015), settlement distances and bleeding volumes of foam can be examined using the following procedure: firstly, clean and dry the testing apparatus’ glass container and glass tube, stainless steel measuring cup, and flat knife. Then, use the measuring cup to take a foam sample and fill the glass container with it. Level the sample slowly along the rim of the measuring cup using the flat knife. Cover the container with an aluminum float and wait for one hour. After that, record the settlement distance value (in mm) accurately to 0.5 mm from the scaled glass container. Next, open the small valve on the glass tube of the testing apparatus and transfer the liquid into a graduated cylinder, recording the bleeding volume (in mL) by reading the liquid volume with accuracy up to 1 mL. The device used to measure settlement distance and bleeding volume of foam is shown in Figure 2.

#### 2.3.2. Performance Test of Foamed Lightweight Soil

**Figure 3:**
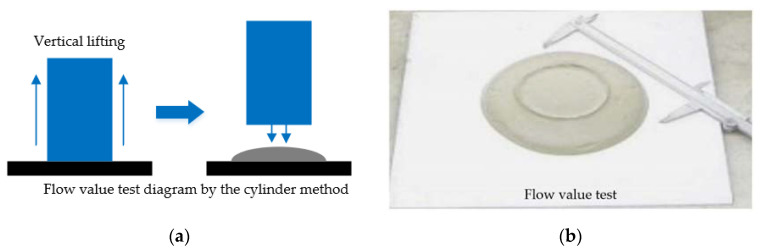
The flow value test diagram: (**a**) Flow value test diagram by the cylinder method; (**b**) flow value test.

In accordance with the Chinese standard “Technical Specification for Foamed Mixture Lightweight Soil Filling Engineering” (CJJ/T 177-2012), the flow value is typically assessed using the cylinder method, utilizing a cylinder with a diameter and height of 80 mm. The following procedure should be followed: clean and dry the hollow cylinder, stainless steel plate, measuring cup, and flat knife before placing the hollow cylinder horizontally on the stainless-steel plate. Use one measuring cup to scoop a sample and carefully transfer the sample into the hollow cylinder without overflowing it. Tap the outside of the hollow cylinder gently with your finger to distribute the sample evenly throughout the cylinder’s entire length. Use a flat knife to slowly level the sample along the upper port plane of the hollow cylinder. Then, lift the hollow cylinder vertically with both hands and let the sample settle for one minute. Finally, measure the maximum horizontal diameter of the sample using a vernier caliper to obtain the actual flow value, illustrated in Figure 3.

The unconfined compressive strength test of foamed lightweight soil grouting material assesses its capacity to withstand external pressure. In line with the Chinese standard “Foamed Concrete Block” (JC/T 1062-2007), a microcomputer-controlled electronic universal testing machine was used in this study to perform uniaxial compression tests on the samples. The unconfined compression strength test value of each sample represents the average of three test results.

Foamed lightweight soil, being a porous cement-based material, forms a large number of pores upon solidification, akin to autoclaved aerated concrete. Accordingly, the water absorption rate of foamed lightweight soil grouting material can be determined, drawing guidance from the “Test Methods of Autoclaved Aerated Concrete” (GB/T 11969-2020). Three specimens are cured for 28 days and transferred to a drying oven set at (60 ± 5) °C and (80 ± 5) °C for 24 h each. The specimens are then dried to a constant weight at (105 ± 5) °C, with their corresponding masses recorded as *M*_0_. After the specimens have been cooled for 6 h at room temperature, they are immersed in a constant temperature water tank maintained at (20 ± 2) °C. Water is added to maintain a height of 1/3 of the specimen for 24 h, followed by adding water to reach 2/3 of the specimen. After an additional 24 h, water is added above 30 mm and kept for another 24 h. Finally, surface moisture is wiped off with a wet cloth, and the specimens are weighed immediately to obtain their mass, recorded as *M*. Using Equation (2), the mass water absorption rate (*W*) of the specimen is calculated.

*W* = (*M* − *M*_0_)/*M*_0_ × 100%, (2)

The water stability of foamed lightweight soil grouting material was investigated using the dry–wet cycle test. After curing for 28 days, specimens were first dried in a (60 ± 5) °C oven for 48 h and then placed in a water tank with a constant temperature of (20 ± 2) °C, where they were fully immersed for 24 h, representing one dry–wet cycle. After repeating this process five times, we recorded the unconfined compressive strength as *q*_u,dw_. The water stability coefficient (*K_r_*), a dimensionless quantity, is defined as the ratio of the unconfined compressive strength value obtained from the dry–wet cycle test to that of the standard cured specimen at 28 days (*q*_u,28_). *K_r_* is computed using Equation (3).

*K_r_* = *q*_u,dw_/*q*_u,28_, (3)

## 3. Results and Discussions

### 3.1. Foam Performance Analysis of Foaming Agent Based on Stability

#### 3.1.1. Effect of Dilution Ratio on Foam Stability

**Figure 4:**
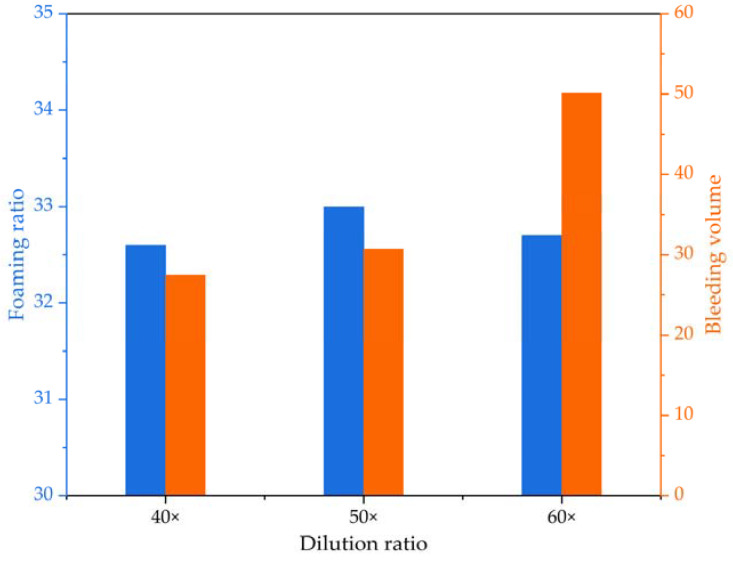
The foaming ratio and bleeding volume at different dilution ratios.

In this study, the foaming agent was diluted at different ratios (i.e., 40×, 50×, and 60×) before producing foam for the foamed lightweight soil grouting material. Following the “Technical Specification for Foamed Concrete Application on Highway” (DB33/T 996-2015), the foam density, foaming ratio, settlement distance, and bleeding volume were tested. Comparative results of foam stability at the three different dilution ratios are presented in Table 3 and Figure 4, including foam density, foaming ratio, settlement distance, and bleeding volume.

The experimental results presented in Table 3 demonstrate a relatively stable foam density for the foaming agent diluted at different ratios, with differences not exceeding 0.4 kg/m^3^. Furthermore, Figure 4 shows that the prepared foam has a stable foaming ratio, with differences not exceeding 0.4 times. However, the one-hour settlement distance of the foam under the three different dilution ratios is negligible, indicating that foam stability cannot be assessed based on the settlement distance at this time. This is likely due to the relatively good foam stability resulting from the foaming agent. Despite some bubbles bursting, the foam mainly comprises neighboring small bubbles merging and connecting into large bubbles, resulting in insignificant sinking and settlement distance. Additionally, the bleeding volume result shown in Figure 4 indicates that as the dilution ratio of the foaming agent increases, the bleeding volume of the prepared foam significantly increases, leading to decreased foam stability.

#### 3.1.2. Foam Stability Improvement

**Figure 5:**
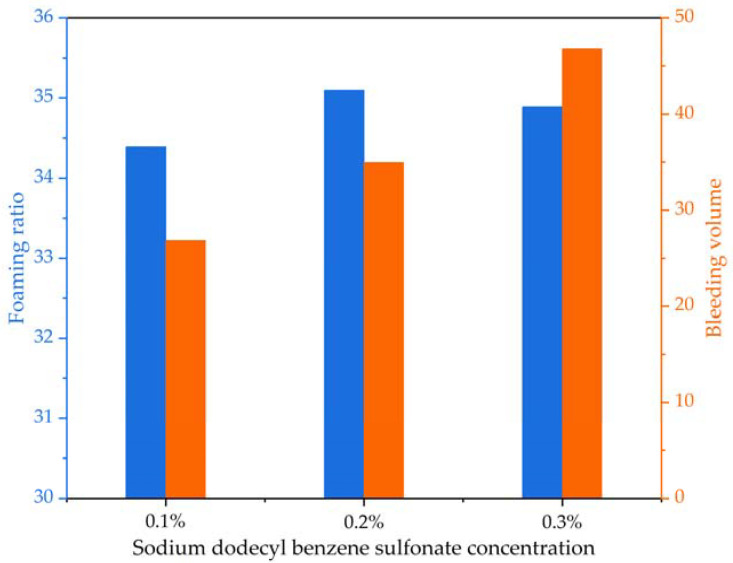
The foaming ratio and bleeding volume at a 40× dilution ratio with different sodium dodecyl benzene sulfonate concentrations.

The analysis indicates that the foaming performance of the foaming agent is better when diluted 40 times. Sodium dodecyl benzene sulfonate is an anionic surfactant known for properties such as emulsification, foaming, and foam stabilization. To evaluate if sodium dodecyl benzene sulfonate is a viable foam stabilizer for the foaming agent, foam stability tests were performed by adding sodium dodecyl benzene sulfonate to the dilution solution. Sodium dodecyl benzene sulfonate powder was added to the foaming agent dilution solution to form solutions with concentrations of 0.1%, 0.2%, and 0.3%, followed by foam preparation. Comparative results of foam stability for the foaming agent at the three different dilution ratios with sodium dodecyl benzene sulfonate, including foam density, foaming ratio, settlement distance, and bleeding volume, are shown in Table 4 and Figure 5, respectively.

A comparison of Figure 4 and Figure 5 reveals that adding 0.2% sodium dodecyl benzene sulfonate to the foaming agent dilution solution increases the foaming ratio. The foaming ratio at a 50× dilution ratio rises to 35.1, indicating that sodium dodecyl benzene sulfonate enhances the foaming ability of the foaming agent. However, comparative analysis among Table 3 and Table 4 as well as Figure 4 and Figure 5 demonstrates that increasing sodium dodecyl benzene sulfonate concentration negatively affects the foam stability generated by the foaming agent. On one hand, the addition of sodium dodecyl benzene sulfonate increases settlement distance, with foam prepared from a foaming agent dilution solution at a dilution rate of 40× with sodium dodecyl benzene sulfonate concentration of 0.3% showing a 1 mm increase in settlement distance after 1 h (Table 4). On the other hand, the bleeding volume from foams prepared from a foaming agent dilution solution with different sodium dodecyl benzene sulfonate concentrations increases to varying degrees (Figure 5). Although sodium dodecyl benzene sulfonate does not significantly alter the properties of the foaming agent, it can function as a foam stabilizer for the foaming agent and improve the foaming ratio to a certain extent without significantly increasing the bleeding volume. Therefore, a sodium dodecyl benzene sulfonate concentration of 0.1% is more appropriate for enhancing the foaming ability of the foaming agent.

### 3.2. Performance Evaluation of Foamed Lightweight Soil Considering Water–Solid Ratios

This study investigates the effect of the water–solid ratio in foamed lightweight soil grouting material on its fundamental physical properties, such as wet density, flow value, and unconfined compressive strength. Moreover, it explores the water absorption and water stability of the foamed lightweight soil grouting material. Foamed lightweight soil grouting material is prepared using a dilution rate of 40× for the foaming agent and a concentration of 0.1% for sodium dodecyl benzene sulfonate. The resulting mixture proportions from Table 2 are used to prepare foamed lightweight soil specimens, which are then tested for their wet density, flow value, unconfined compressive strength, water absorption, and water stability.

#### 3.2.1. Effect of Water–Solid Ratio on Flow Value

**Figure 6:**
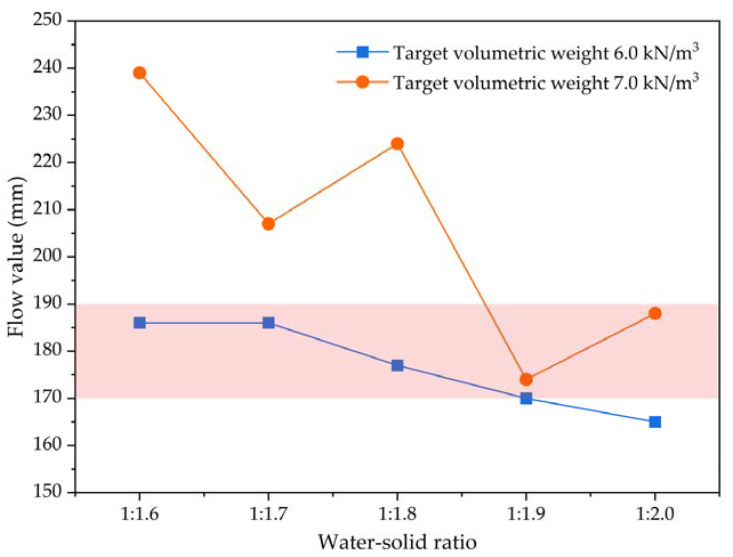
The flow value results of foamed lightweight soil at various water–solid ratios.

Flow value is used to measure the fluidity of foamed lightweight soil grouting material. The foamed lightweight soil grouting material has good fluidity and can achieve a self-compacting construction state without rolling and vibration. According to the Chinese standard “Technical Specification for Foamed Mixture Lightweight Soil Filling Engineering” (CJJ/T 177-2012), the flow value results are tested using the cylinder method, as shown in Table 5 and Figure 6.

Based on the experimental results, at a target volumetric weight of 6.0 kN/m^3^, an increase in solid proportion (ratio of cement) in the water–solid ratio parameter of foamed lightweight soil grouting material leads to a decrease in its flow value. This suggests that decreasing the water content has a significant impact on fluidity within this volumetric weight range, which aligns with existing literature [26]. However, at a target volumetric weight of 7.0 kN/m^3^, the flow value of the foamed lightweight soil grouting material fluctuates with the water–solid ratio, unlike the pattern observed at 6.0 kN/m^3^. Under higher volumetric weights, bubbles in the foamed lightweight soil break and gather, forming large pores that negatively affect fluidity, resulting in a downward trend of fluctuation. When the water–solid ratio is constant, increasing the amount of cement paste improves the fluidity of foamed lightweight soil grouting material with a target volumetric weight of 7.0 kN/m^3^ compared to that with a target volumetric weight of 6.0 kN/m^3^. According to the Chinese standard “Technical Specification for Design and Construction of Cast-in situ Foamed Lightweight Soil Subgrade” (TJG F1001-2012), the flow value of foamed lightweight soil grouting material in physical engineering should be between 170 and 190 mm. Thus, it can be concluded that a water–solid ratio of 1:1.6–1:1.9 meets the flow value requirement of foamed lightweight soil grouting material at a target volumetric weight of 6.0 kN/m^3^. Similarly, a water–solid ratio of 1:1.9–1:2.0 meets the flow value requirement of foamed lightweight soil grouting material at a target volumetric weight of 7.0 kN/m^3^.

#### 3.2.2. Effect of Water–Solid Ratio on Unconfined Compression Strength

**Figure 7:**
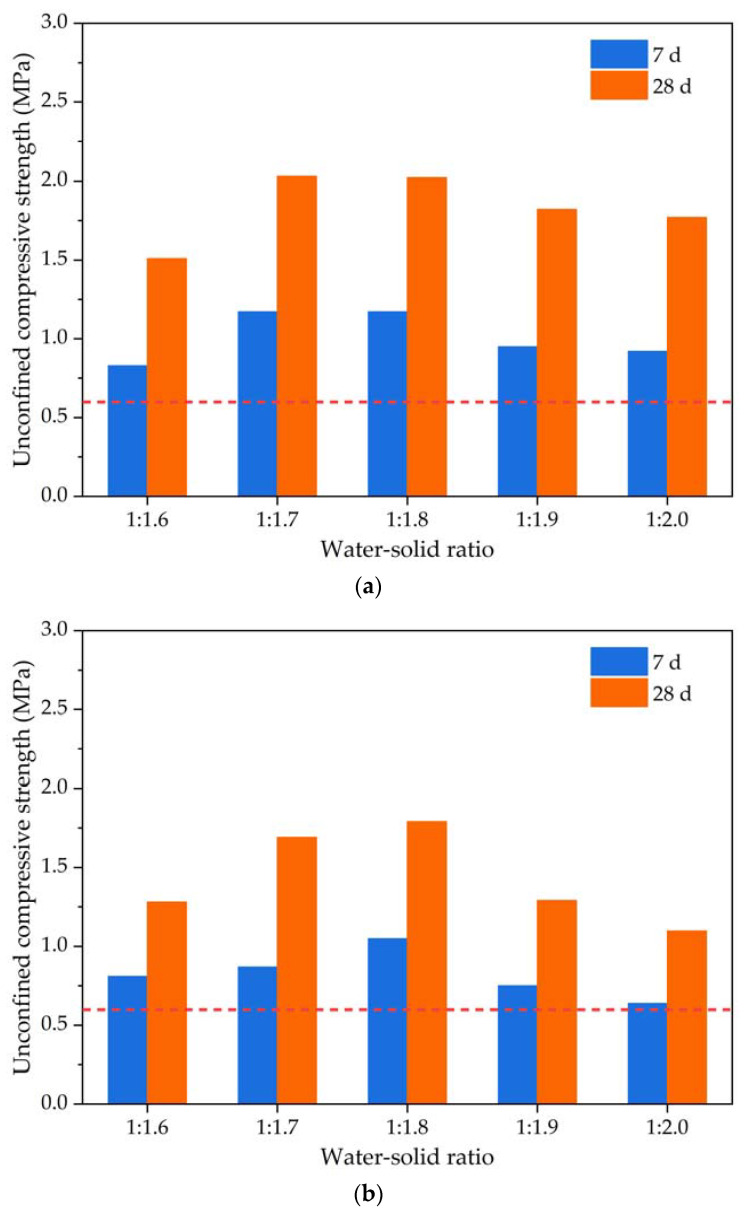
The unconfined compressive strength test results of the foamed lightweight soil grouting material: (**a**) Foamed lightweight soil with target volumetric weight of 6.0 kN/m^3^; (**b**) foamed lightweight soil with target volumetric weight of 7.0 kN/m^3^.

Unconfined compressive strength is a crucial indicator of the mechanical strength properties of foamed lightweight soil grouting material. It denotes the maximum vertical compressive stress that an unconfined foamed lightweight soil specimen can withstand. Due to the material’s high porosity, it is vital to ensure that the compressive strength of foamed lightweight soil grouting material meets the necessary engineering construction standards at low densities. This concern is particularly relevant in practical applications. The unconfined compressive strength test results of foamed lightweight soil grouting material prepared for two distinct groups with target volumetric weights of 6.0 kN/m^3^ and 7.0 kN/m^3^ are illustrated in Figure 7.

The unconfined compressive strength test results in Figure 7 indicate that the unconfined compressive strength of the foamed lightweight soil specimens cured for 7 and 28 days increases initially and then decreases as the water–solid ratio changes from 1:1.6 to 1:2.0. The maximum unconfined compressive strength values for the specimens cured for 7 and 28 days occur when the water–solid ratio is between 1:1.7–1:1.8. The reduction in strength could be due to the formation of interconnected pores inside the foamed lightweight soil grouting material caused by the evaporation of water during the hardening process when the solid proportion in the water–solid ratio is small (i.e., the water proportion is larger). On the other hand, a large solid proportion of foamed lightweight soil grouting material could increase defects, leading to a decrease in the unconfined compressive strength due to increased friction between foam and cement paste and uneven distribution of foam in the paste. In addition, the unconfined compression strength of foamed lightweight soil materials at different target volumetric weights indicates that higher volumetric weight and cement content result in smaller strength than lower volumetric weight and cement content. This could be attributed to the generation of more internal stress in foamed lightweight soil materials with high cement content and volumetric weight, resulting in cracks and defects that reduce strength performance. Furthermore, high-volumetric weight cement materials could have larger porosity, affecting the compactness and strength of foamed lightweight soil materials. Conversely, low-cement content and low-volumetric weight foamed lightweight soil materials can reduce internal stresses and porosity effectively and improve material compactness, thereby enhancing their strength performance.

According to the Chinese standard “Technical Specification for Design and Construction of Cast-in situ Foamed Lightweight Soil Subgrade” (TJG F1001-2011), it is stipulated that for high-grade highways, when the top surface of the foamed lightweight soil subgrade is more than 0.8 m away from the bottom surface of the road, the 28-day unconfined compressive strength of the foamed lightweight soil should not be less than 0.6 MPa. Therefore, the abovementioned mix proportions of foamed lightweight soil grouting material with the target volumetric weight of 6.0 kN/m^3^ and 7.0 kN/m^3^ meet the strength requirements. The foamed lightweight soil specimens with the target volumetric weight of 6.0 kN/m^3^ are prepared for the water absorption and water stability.

#### 3.2.3. Effect of Water–Solid Ratio on Water Absorption

**Figure 8:**
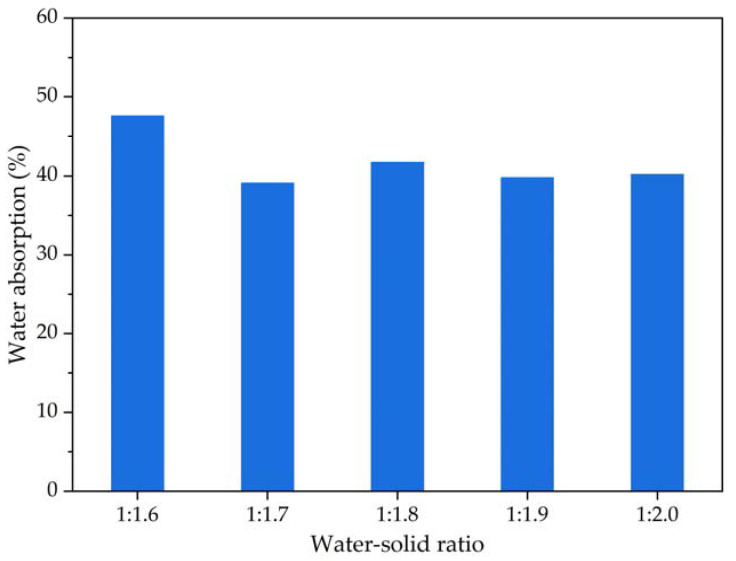
The mass water absorption results of foamed lightweight soil at various water–solid ratios.

In this study, the water absorption is represented by water absorption rate, which refers to the ability of foamed lightweight soil grouting material to absorb water under the soaking state. Referring to the “Test Methods of Autoclaved Aerated Concrete” (GB/T 11969-2020), the water absorption rate results of foamed lightweight soil grouting material are tested. The measured mass water absorption of foamed lightweight soil grouting material is shown in Figure 8.

Figure 8 shows that the water absorption rate of the foamed lightweight soil grouting material varies with changes in the water–solid ratio. At a water–solid ratio of 1:1.6, the water absorption rate is the highest at 47.6%. However, when the water–solid ratio is between 1:1.7 and 1:2.0, the water absorption rate is lower than that of foamed lightweight soil grouting material with a water–solid ratio of 1:1.6, and the water absorption rate of foamed lightweight soil grouting material with different water–solid ratios changes slightly. The internal pore connectivity of the foamed lightweight soil grouting material affects its water absorption rate. A greater number of connected pores results in a higher water absorption rate. Therefore, the similar water absorption rates of the foamed lightweight soil grouting material with water–solid ratios of 1:1.7–1:2.0 indicate similar internal pore conditions. The higher water absorption rate of the foamed lightweight soil grouting material with a water–solid ratio of 1:1.6 suggests that a high water proportion in the water–solid ratio can promote the formation of connected pores inside the foamed lightweight soil grouting material.

#### 3.2.4. Effect of Water–Solid Ratio on Water Stability

**Figure 9:**
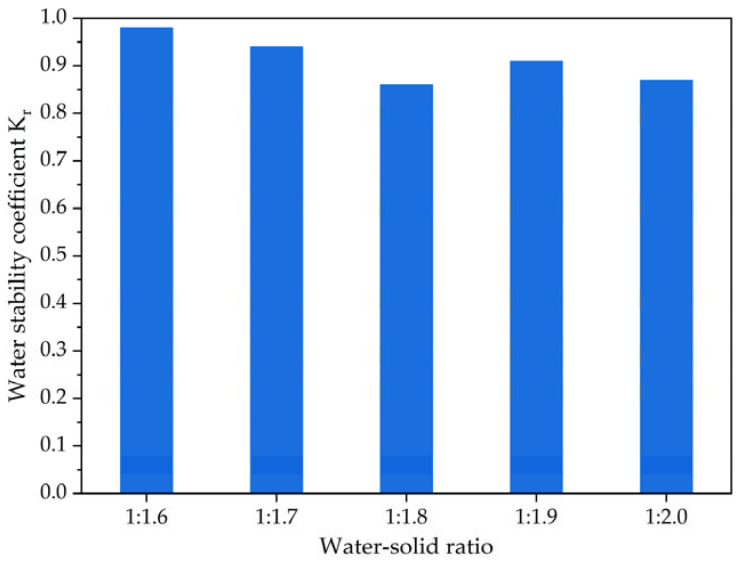
The water stability coefficient results of foamed lightweight soil at various water–solid ratios.

Water stability describes a subgrade’s resistance to the adverse effects of water after being invaded by it. The water stability coefficient typically characterizes a soil’s water stability, with higher coefficients indicating better performance. In areas with frequent rainfall, roads are often exposed to water and erosion. If foamed lightweight soil grouting material is used as a filling or grouting material for subgrades, its water stability performance must meet high requirements. Therefore, this study uses the dry–wet cycle test to investigate the water stability of the foamed lightweight soil grouting material, and the calculated water stability coefficient results are presented in Figure 9.

As shown in Figure 9, the dry–wet cycles results in a decrease in the unconfined compressive strength of foamed lightweight soil grouting material. This is mainly due to the fact that under dry–wet cycle conditions, the pore wall inside the foamed lightweight soil grouting material is constantly subjected to stress damage, resulting in a decrease in its unconfined compressive strength. From the water stability coefficient results of foamed lightweight soil grouting material at various water–solid ratios, it can also be seen that under the five mixing ratios, the water stability coefficients of foamed lightweight soil grouting material are all greater than 0.85, indicating that although there are many pores inside the foamed lightweight soil grouting material, the corresponding water stability is good. The water absorption results also showed that despite the changing water–solid ratio, the internal pore conditions are relatively stable, leading to good water stability. In future studies, more dry–wet cycles will be carried out to investigate the durability evaluation of foamed lightweight soil, which would provide more systematic research conclusions.

## 4. Conclusions

This study investigates the preparation and performance of foamed lightweight soil grouting material for goaf treatment. Initially, foam stability is analyzed by testing the density, foaming ratio, settlement distance, and bleeding volume of foam at different foaming agent dilution ratios. Furthermore, the impact of the water–solid ratio on the basic physical properties of foamed lightweight soil grouting material is examined. Additionally, the water absorption and water stability of the material are studied. Based on the study’s findings, the following conclusions can be drawn:

(1) The dilution ratio of the foaming agent affects foam stability. The settlement distance of foam does not vary significantly for different dilution ratios, with no more than a 0.4 times difference in the foaming ratio. However, bleeding volume is positively correlated with the dilution ratio of the foaming agent. The bleeding volume of the foaming agent at a 60× dilution ratio is approximately 1.5 times that at a 40× dilution ratio, reducing the foam stability. Furthermore, an appropriate amount of sodium dodecyl benzene sulfonate is suitable for enhancing the foaming capacity of the foaming agent and stabilizing the foam.

(2) The water–solid ratio affects the flowability of foamed lightweight soil. The suitable water–solid ratios for producing a target volumetric weight of 6.0 kN/m^3^ and 7.0 kN/m^3^, while satisfying the required flow value of 170~190 mm, range between 1:1.6~1:1.9 and 1:1.9~1:2.0, respectively.

(3) The unconfined compressive strength of foamed lightweight soil initially increases and then decreases after curing for 7 days and 28 days as the proportion of solids in the water–solid ratio increases. The maximum value is achieved at a water–solid ratio of 1:1.7~1:1.8. Additionally, the unconfined compressive strength values at 28 days are approximately 1.5~2 times higher than those at 7 days.

(4) Water absorption tests indicate that excessive water in the water–solid ratio leads to connected pores inside the foamed lightweight soil, resulting in a higher water absorption rate. Thus, a water–solid ratio of 1:1.6 should be avoided.

(5) Foamed lightweight soil experiences reduced unconfined compressive strength under dry–wet cycling conditions, but the rate of strength loss is minimal. Additionally, foamed lightweight soil prepared with various water–solid ratios satisfies the durability requirements under dry–wet cycling conditions.

In summary, foamed lightweight soil grouting material with a target density of 6.0 kN/m^3^ demonstrates superior flowability, unconfined compressive strength, water absorption, and water stability when the water–solid ratio is set between 1:1.7~1:1.8. Further research involving micro analysis and modification mechanisms should be conducted to facilitate a more systematic evaluation of foamed lightweight soil in future studies.

## Figures and Tables

**Table 1:** Basic properties of cement in this study.

Item	Density (kg/m^3^)	Standard Consistence (%)	Initial Setting Time (min)	Final Setting Time (min)	Composition (%)				
					SiO_2_	CaO	Al_2_O_3_	Fe_2_O_3_	LOI
Result	3117	28.7	390	535	21.14	60.43	6.11	2.54	1.02

**Table 2:** The mix proportion of foamed lightweight soil grouting material.

No.	Target Volumetric Weight (kN/m^3^)	Water–Solid Ratio	Cement (kg/m^3^)	Water (kg/m^3^)	Foam (kg/m^3^)
A0	6.0	1:1.6	356	223	21
A1		1:1.7	364	214	21
A2		1:1.8	372	207	21
A3		1:1.9	379	199	21
A4		1:2.0	386	193	22
B0	7.0	1:1.6	419	261	19
B1		1:1.7	429	252	19
B2		1:1.8	437	243	19
B3		1:1.9	446	234	20
B4		1:2.0	453	226	20

**Table 3:** The foam density and settlement distance at different dilution ratios.

Dilution Ratio	Foam Density (kg/m^3^)	Settlement Distance (mm)
40×	30.7	0
50×	30.3	0
60×	30.3	0

**Table 4:** The foam density and settlement distance at a 40× dilution ratio with different sodium dodecyl benzene sulfonate concentrations.

Concentration	Foam Density (kg/m^3^)	Settlement Distance (mm)
0.1%	30.9	0
0.2%	30.2	0
0.3%	29.7	1

**Table 5:** The flow value results of foamed lightweight soil at various water–solid ratios.

No.	Target Volumetric Weight (kN/m^3^)	Water–Solid Ratio	Flow Value (mm)	Wet Density (kN/m^3^)
A0	6.0	1:1.6	186	6.35
A1		1:1.7	186	6.64
A2		1:1.8	177	6.34
A3		1:1.9	170	6.58
A4		1:2.0	165	6.26
B0	7.0	1:1.6	239	6.58
B1		1:1.7	207	7.10
B2		1:1.8	224	7.30
B3		1:1.9	174	6.84
B4		1:2.0	188	6.22

## Data Availability

Not applicable.
